# PathwAX II: network-based pathway analysis with interactive visualization of network crosstalk

**DOI:** 10.1093/bioinformatics/btac153

**Published:** 2022-03-10

**Authors:** Christoph Ogris, Miguel Castresana-Aguirre, Erik L L Sonnhammer

**Affiliations:** Department of Biochemistry and Biophysics, Stockholm University, Science for Life Laboratory, 17121 Solna, Sweden; Institute of Computational Biology, Helmholtz Center Munich, 85764 Neuherberg, Germany; Department of Biochemistry and Biophysics, Stockholm University, Science for Life Laboratory, 17121 Solna, Sweden; Department of Biochemistry and Biophysics, Stockholm University, Science for Life Laboratory, 17121 Solna, Sweden

## Abstract

**Motivation:**

Pathway annotation tools are indispensable for the interpretation of a wide range of experiments in life sciences. Network-based algorithms have recently been developed which are more sensitive than traditional overlap-based algorithms, but there is still a lack of good online tools for network-based pathway analysis.

**Results:**

We present PathwAX II—a pathway analysis web tool based on network crosstalk analysis using the BinoX algorithm. It offers several new features compared with the first version, including interactive graphical network visualization of the crosstalk between a query gene set and an enriched pathway, and the addition of Reactome pathways.

**Availability and implementation:**

PathwAX II is available at http://pathwax.sbc.su.se.

**Supplementary information:**

Supplementary data are available at *Bioinformatics* online.

## 1 Introduction

Advances in modern molecular biology have led to a multitude of high throughput techniques to discover altered expression levels between different conditions, for example, healthy and diseased cells. The resulting list of significantly altered genes usually gives few biological insights on its own, and therefore researchers often move on to determine which pathways are activated (Ooi *et al.*, 2010). The oldest and most widely used technique is based on determining statistically significant overlap between a query gene set and a pathway (Huang *et al.*, 2009), which however have been shown to have both a high false-positive rate (Gatti *et al.*, 2010) and low detection rates (Ogris *et al.*, 2017). Overlap-based analysis can be enhanced by functional class scoring (Subramanian *et al.*, 2005; Tarca *et al.*, 2012) or pathway topology (Draghici *et al.*, 2007) methods when gene expression data are available, but this is not always the case.

In contrast to overlap-based methods, state-of-the-art network-based methods have the ability to find significant associations between gene sets and pathways even in the absence of shared genes. This is achieved by evaluating the enrichment of the interactions, crosstalk, between a pathway and a gene set within a biological network like FunCoup (Ogris *et al.*, 2018), GeneMania (Zuberi *et al.*, 2013) or STRING (Szklarczyk *et al.*, 2019). Even though benchmarks show that network-based methods have a clear advantage (Ogris *et al.*, 2017), their use is still not widespread among the community. This might be due to the fact that these methods often suffer from low usability and increased compute time, which results from processing genome-wide functional association networks.

To counter this issue, we developed PathwAX (Ogris *et al.*, 2016), which is a web server for online pathway annotation which uses the network-based BinoX (Ogris *et al.*, 2017) algorithm. The method identifies enriched pathways by estimating the amount of crosstalk within a network, between a set of pathway genes and gene set of interest, and compares it to the amount of crosstalk expected by chance. By preprocessing the input networks, PathwAX reduces the computation time by orders of magnitude and provides a rapid online network-based pathway analysis for a predefined set of pathways.

Here, we present PathwAX version II. In addition to the version I functionality, it is equipped with a new interactive graphical network viewer for visualizing details of the connections between the query and pathway genes, which allows the user to inspect which genes and connections that underlie the observed crosstalk. This can for instance be used to see if the crosstalk is spread out between many genes or dominated by a small number of hubs, or to identify which pathway genes are connected to the largest number of query genes. It also features updated networks and KEGG pathways, and the addition of Reactome pathways (Jassal *et al.*, 2019).

## 2 Results

See Supplementary Materials for information on the implementation and more examples of how to use PathwAX II.

We illustrate the usage of PathwAX II by annotating the gene list LOPEZ_MESOTHELIOMA_SURVIVAL_WORST_VS_BEST_UP (López-Ríos *et al.*, 2006) from MSigDB (Liberzon *et al.*, 2011). It comes from a study of 99 pleural mesothelioma patients that produced a list of 14 upregulated genes that cause the highest lethality. Annotating this gene list with KEGG pathways using PathwAX II results in 71 enriched and one depleted pathway at Family-Wise Error Rate (FWER) < 0.05 (Supplementary Fig. S1). Compared with the previous release, PathwAX II identifies many new enrichments. For example, ranked 4 is the PI3K-Akt signaling pathway at FWER = 6.46×10^-8^, which has been shown to impact mesothelioma survival (Bonelli *et al.*, 2017; Zhou *et al.*, 2014).

Using the Reactome database which was added in PathwAX II, it identifies 35 enriched and six depleted pathways at a significance threshold of FWER < 0.05. None of these pathways would have been detected using conventional overlap-based methods such as DAVID (Huang *et al.*, 2009) since they do not contain more than one query gene. Here, translocation of GLUT4 to the plasma membrane is the most significant enriched one at FWER = 6.44×10^-7^. The glucose transporter GLUT4 has been shown to be transcriptionally repressed by the p53 tumor suppressor, which is an important protein in cell cycle control and apoptosis and has been linked to PI3K-Akt signaling pathway in cancer (Calvo *et al.*, 2010). The second most significant pathway is Platelet degranulation at FWER = 1.51×10^-6^. Platelets are important for angiogenesis, inflammation, wound healing, hemostasis and cancer formation. Cancer cells with access to the bloodstream trigger platelet-mediated recognition, which leads to a higher survival and spread of cancer cells (Menter *et al.*, 2014).

**Fig. 1.**
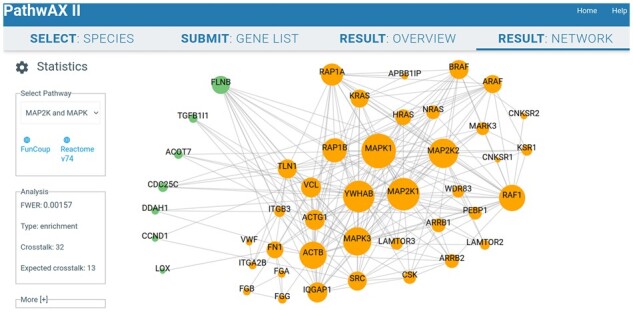
Interactive network-based pathway analysis with PathwAX II. This example visualizes the network crosstalk between the query gene set LOPEZ_MESOTHELIOMA_SURVIVAL_WORST_VS_BEST_UP from MSigDB in green (separated to the left), and Reactome's MAP2K and MAPK activation pathway in orange (separated to the right). See Supplementary Figure S1 for the optional grid layout of the graph and the overview table of pathways matching the query

A key pathway in cancer, MAP2K and MAPK activation, found by PathwAX II at FWER = 1.57×10^-3^, is linked to regulation of cell proliferation, differentiation, migration and apoptosis (Dhillon *et al.*, 2007). It has been shown that inhibiting MAPK reduces the survival of mesothelioma cells (Roth *et al.*, 2009). The Result: Network view of the query genes and the MAP2K and MAPK activation pathway can be displayed by PathwAX II either as a force field layout (Fig. 1) or grid layout (Supplementary Fig. S1B) that both allow the user to move around nodes to optimize visibility. This network view in PathwAX II adds useful information about the crosstalk that was not available in version I. For instance, one can see which pathway genes have the most links to the query genes. In this case, MAPK1 and YWHAB top the list with four linked query genes each, of which three are common. Conversely, one can see which pathway genes are linked to any query gene, which also was not possible in version I. In this example, it is notable that 16 of the 40 pathway genes are linked to the query gene set, indicating a widespread crosstalk. Moreover, the pathway genes with most links to the query set: MAPK1, MAP2K1, YWHAB, MAP2K2 and MAPK3 are known to be highly expressed in various sorts of cancer (Uhlen *et al.*, 2015), which further highlights the connection to this cancer-derived query set. In total, the query-pathway crosstalk is 32 links while one would only expect 13 links by chance. Even though these gene sets do not share a common gene, and would thus be undetectable with overlap-based methods, this level of crosstalk provides evidence to classify MAP2K and MAPK activation as significantly enriched.

## Data availability

The data underlying this article are available in the FunCoup, KEGG, and Reactome databases.

## Supplementary Material

btac153_Supplementary_Data
