# Supplementary material for: PathwAX II: network-based pathway analysis with interactive visualization of network crosstalk
Source: Bioinformatics. 2022 Mar 10;38(9):2659–60. doi: 10.1093/bioinformatics/btac153 (PMC9048662; doi:10.1093/bioinformatics/btac153)
Supplement: btac153_Supplementary_Data [file btac153_supplementary_data.pdf]

# Supplementary Materials

# PathwAX II: Network-based pathway analysis with interactive visualization of network crosstalk

Christoph Ogris[12*]†, Miguel Castresana-Aguirre[1]†, and Erik L.L. Sonnhammer[1]

## IMPLEMENTATION

### INTERACTIVE NETWORK-BASED PATHWAY ANALYSIS

PathwAX II consists of tabs guiding the user through the analysis; *Select: Species*, *Submit: Gene List*, *Result: Overview,* and the new interactive *Result: Network* tab.

The *Result: Overview* tab now features the option to select different methods for multiple testing correction - Bonferroni, Benjamini-Hochberg or none. Also, one can now expand the new panel *More* to export and save the results as a csv file.

While the *Result: Overview* tab still serves the purpose of giving a summary of all found pathways, the new *Result: Network* tab displays and visualizes the network crosstalk between the query set and one pathway, as well as additional statistics of the enrichment/depletion and crosstalk.

An enriched/depleted pathway can be either selected in the *Result: Overview* tab by clicking on a row of the pathway table or directly in the upper left panel *Select Pathway* in the *Result: Network* tab. When a pathway is selected, PathwAX fetches and displays the main statistical facts of the analysis in the *Analysis* panel. Additional information can be displayed by expanding the panel with *More*. Simultaneously PathwAX II renders a dynamic graphical representation of a network consisting of query and pathway genes as nodes. Here the size of a node refers to its degree and the color indicates if a node belongs to the pathway set (orange) or query set (green), or if it is a shared node (purple) present in both sets. To focus on a single node and its neighbours, one can move the mouse over the node of interest which hides all unconnected nodes. For small networks, PathwAX II uses a force field visualization style to display the network. In cases when the graph contains more than 1000 links, the force field tends to become unreadable and the visualization style is switched to gridview. In gridview mode the query nodes are displayed first, followed by shared nodes, and then by pathway nodes. The nodes within each group are ordered by their link degree. Hub nodes with high degree are displayed first, while single stand alone nodes are listed last. The visualization style can be chosen manually in the *Graph Style* panel if the network has less than 1000 links.

Expanding the *Visualization* section's *More* panel gives the user the possibility to alter the Pathway node labels. By default the viewer displays these as UniProt accessions. Depending on the species, the label can be changed to *Ensembl, Gene Symbol, NCBI, FlyBase, WormBase, PomBase, ZFIN, or TairLocus* accessions. Additionally one can also switch to no label at all or the node degree in the subnetwork, reflecting topological network properties.

PathwAX II provides a direct link to the FunCoup website requesting the subnetwork of query and pathway genes for further analysis. FunCoup will highlight query genes with black borders but disregard any pathway genes which are not connected to the query. Finally a link to the used pathway database provides more information about the pathway under investigation.

**STATISTICAL METHOD**

PathwAX II uses the same statistical model as PathwAX I, *i.e.* the BinoX algorithm (Ogris et al., 2017). Briefly, the network is randomized 1000 times and using these, the average number of connections k' for each query-pathway pair is calculated and used as an estimate of the expected connections within a randomised environment, E(k'). The binomial distribution is used to calculate the statistical significance of the observed connections k. The alternative binomial distribution depends on n', the maximum possible connections between gene set and pathway, and p', the probability of observing k'. Here p' is approximated by E(k')/n'. Bonferroni correction is by default used to account for multiple testing to calculate FWER values, and Benjamini-Hochberg corrected FDR values can optionally be used. The performance of the algorithm used in PathwAX II has previously been assessed (Ogris *et al.*, 2017).

**DATA**

## Networks

The underlying networks of PathwAX are the key elements of the analysis. Here, PathwAX II relies on the networks provided by FunCoup, a database for genome wide functional association networks (Ogris *et al.*, 2018). FunCoup uses a redundancy weighted Bayesian integration approach to combine ten different evidence types of functional association; mRNA co-expression, protein co-expression, protein interaction, phylogenetic profile similarity, genetic interaction profile similarity, subcellular co-localization, domain interaction, quantitative mass spectrometry, co-microRNA regulation, and shared transcription factor binding. FunCoup contains networks for *Homo sapiens* and 16 model organisms making it one of the most comprehensive network resources available. FunCoup links with a confidence score > 0.8 were used to ensure high quality. Across all networks, PathwAX II covers 116 581 genes and 11 355 693 interactions between them (see Table 1).

All species networks were shuffled 1000 times to generate random representations. The shuffling procedure was done by using the Link Assignment and Second Order Conservation method implemented in BinoX (Ogris *et al.*, 2017). This method preserves the network's second order topological properties and degree distribution.

## Pathways

In PathwAX II we updated the KEGG pathway data to release 94.1. PathwAX II contains 5 269 pathways for 17 species (see Figure S2). In sum the KEGG pathways represent 65 215 unique genes which is an increase of 20 857 genes compared to version 1. In addition to KEGG, PathwAX II also supports pathway annotation using Reactome pathways v74. Unfortunately there is no Reactome data available for *Ciona intestinalis*, *Escherichia coli,* and *Bacillus subtilis*. For the other species, the majority of Reactome pathways on the lowest level are very specific. 46% of these pathways consist of less than 10 genes, once we map them to FunCoup networks. To avoid very small pathways, we resolved Reactome's

hierarchy by collapsing lower level pathways below a certain pathway size to their parents until obtaining an average pathway size similar to KEGG pathways for each species. The minimum pathway size for collapsing child nodes varies between species. This reduced Reactome from 1166 pathways on average per species to 155.

**SERVER INFORMATION AND SOFTWARE IMPLEMENTATION**

The web server is a virtual machine running CentOS 6.9 with 2 GB RAM and 2 Intel Xeon E5-2630v2 2.60 GHz cores. The PathwAX client uses the JavaScript libraries D3 v4 (Bostock *et al.*, 2011), jQuery v2.1.4 and materialize. On the server all processes are implemented in a python interface.

## PathwAX ANALYSIS OF IMMATURE NEUTROPHILS

In this example we investigate the gene set MARTINELLI_IMMATURE_NEUTROPHIL_UP, also obtained via the MSigDB v3.0 collection (Liberzon *et al.*, 2011) to demonstrate PathwAX's analytical potential. This set contains 11 genes up-regulated in immature neutrophils relative to mature neutrophils, and was used to investigate the process of neutrophil maturation and extracellular traps during differentiation (Martinelli *et al.*, 2004).

To analyse this gene set we submitted the 11 up-regulated genes to PathwAX with KEGG as reference pathway database (default). Based on this input, PathwAX identifies 22 significantly enriched KEGG pathways at FWER (Family-Wise Error Rate) < 0.05 (Figure S3A). Among these, only five pathways share more than one gene with the input gene set, hence an overlap-based method such as DAVID (Huang *et al.*, 2009) would be unable to detect the remaining 17 pathways. The KEGG pathway classes show that a majority of the detected KEGG pathways, 15, are classified as *Human Disease* pathways, two as *Cellular Processes*, one as *Environmental Information Processing* and four as *Organismal Systems.*

The highest ranked pathway is *Systemic lupus erythematosus* (SLE) with an FWER of $2.62*10^{-84}$. The link between SLE and immature neutrophils has previously been observed (Bennett *et al.*, 2003). Interestingly most of the MARTINELLI_IMMATURE_NEUTROPHIL_UP genes are listed among those molecules which are expelled with neutrophil extracellular traps to tackle bacteria and are associated with SLE (Salemme *et al.*, 2019). This may also explain why PathwAX finds the *Staphylococcus aureus infection* pathway as enriched (FWER = $1.15*10^{-28}$). There are 3 overlapping genes to this pathway - DEFA1 and DEFA4 from the defensin protein family which are known antimicrobials found in neutrophils, and the CAMP gene which encodes the Cathelicidin antimicrobial peptides LL-37 and FALL-39. Thanks to the network viewer of PathwAX, it can be observed that there are 4 main network modules in the crosstalk between the gene set and the *Staphylococcus aureus infection* pathway (Figure S3B). One module contains the defensins DEFA1 and DEFA4 as overlapping genes between the query and the pathway, but also other members of the same family such as DEFA1B and DEFA3. The same module further contains the genes AZU1, which encodes CAP37 protein, a known

opsonin for *S. aureus* (Heinzelmann *et al.*, 1998), ELANE, CTSG, and MPO. All these genes are expressed in azurophilic granules (McKay *et al.*, 1999), which could explain why they are also found in the same module.

PathwAX further finds significant enrichment of the *NOD-like receptor signaling* pathway (FWER = $3.28*10^{-28}$), whose role in the neutrophil immune responses is not yet characterized, but a study (Ekman and Cardell, 2010) suggests that NLRs may be a previously unknown pathway for neutrophil activation. Further, the significantly enriched *Phagosome* pathway (FWER = $7.69*10^{-9}$) is supported by the reduced phagocytic capacity of immature neutrophils compared to matured ones (Mackey *et al.*, 2019).

## DISCUSSION

We have described the PathwAX II web server, a versatile interactive tool for online pathway annotation based on network crosstalk, and showed how it can be used to discover significant pathway enrichment even in the absence of shared genes. The new query-pathway network viewer directly visualizes the genes involved in the crosstalk as well as the network topology of the query and pathway gene sets.

Overall PathwAX II shows an increased detection rate which spawns the legitimate question of whether this high detection rate is followed by an increased false positive rate. To investigate this, we generated and analyzed 100 random gene sets of 100 genes for *Homo sapiens* with PathwAX I and PathwAX II. A false positive was counted for each significantly enriched or depleted pathway. We found that at FWER < 0.05, PathwAX II had a median false positive rate of 0.026, compared to 0.035 for PathwAX I.

There exists a wide spread of resources providing pathway information like GeneOntology (Ashburner *et al.*, 2000), OmniPath (Türei *et al.*, 2016), PathwayCommons (Rodchenkov *et al.*, 2020), WikiPathways (Slenter *et al.*, 2018), Panther (Mi *et al.*, 2017), Reactome (Jassal *et al.*, 2019; Croft *et al.*, 2014), and KEGG (Kanehisa *et al.*, 2016). Pathway ontology definitions vary between databases, making the overlap between them small (Domingo-Fernández *et al.*, 2019), resulting in different pathway enrichment results (Mubeen *et al.*, 2019). Evaluating KEGG pathway classes shows that it has a bias towards metabolic pathways. Unfortunately KEGG does not define a separate class for signaling pathways. To account for this deficiency we extended PathwAX by including Reactome pathways. In Reactome the biggest class is the *Signal Transduction* consisting of 45 pathways (in human). Reactome and KEGG developers have different curation approaches, making the distribution of pathways data more heterogeneous. Both databases rely on manually curated pathway data extracted from experimental data, which depends on human resources. This may also cause the same pathway to be named differently in different databases (Chowdhury and Sarkar, 2015). KEGG tends to have broad terms while Reactome has a hierarchical structure of pathways, allowing very specific and small entries. When introducing Reactome it became apparent that the large number of small and overlapping pathways was detrimental for the analysis. Therefore we truncated the reactome hierarchy to include terms with a similar level of specificity as in KEGG.

As in the previous release, PathwAX II only allows submitting up to 400 genes. While this might seem like an arbitrary limit, it is partly motivated by the maximum number of discernable genes as columns in the overview section. For annotating bigger gene sets we refer to the stand alone tool BinoX which is freely available under https://sonnhammer.org/BinoX.

A.

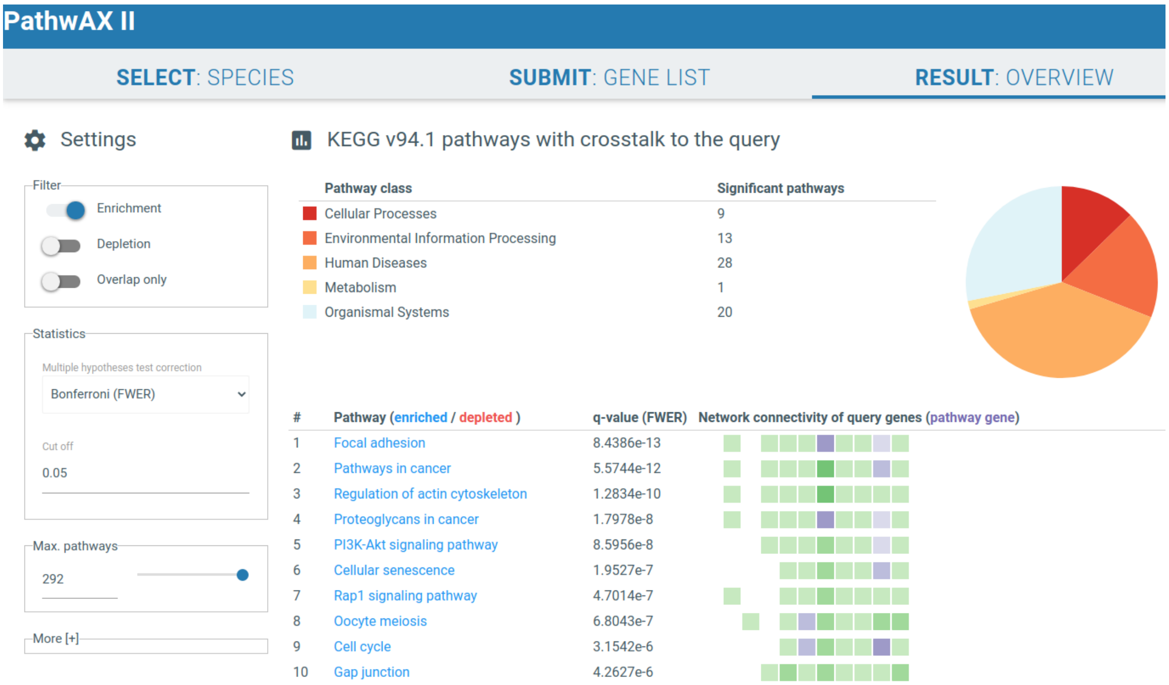


B.

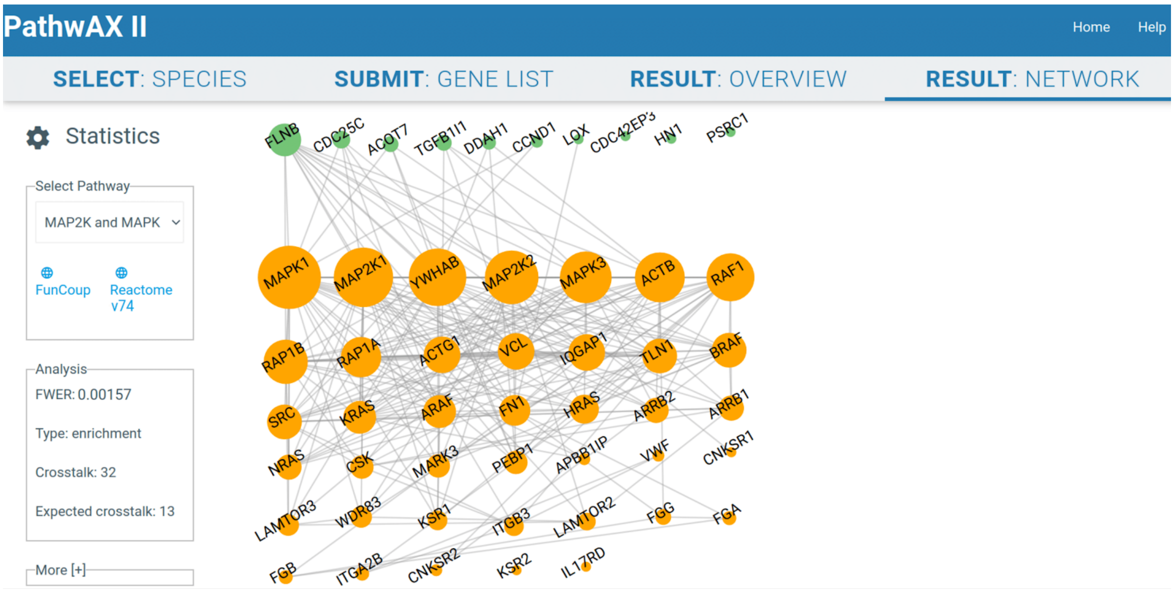


**Figure S1.** PathwAX II results for the 11 genes in the human gene set LOPEZ_MESOTHELIOMA_SURVIVAL_WORST_VS_BEST_UP in MSigDB. **(A)** The table lists all enriched (blue) and depleted (red) pathways (none were detected in the top 10) for the query that are significant for the chosen cutoff (only top part shown). The results are sorted by increasing FWER. To the right is a matrix showing network connections between query genes and each pathway. Each gene is shown as a coloured box and mouseover shows its number of links to the pathway. Green boxes represent query genes linked to the pathway and purple boxes indicate genes which are part of the pathway. Darker shades indicate higher connectivity. **(B)** The network grid layout against Reactome's *MAP2K and MAPK activation* pathway. Grid layout is enforced if the graph contains more than 1000 links.

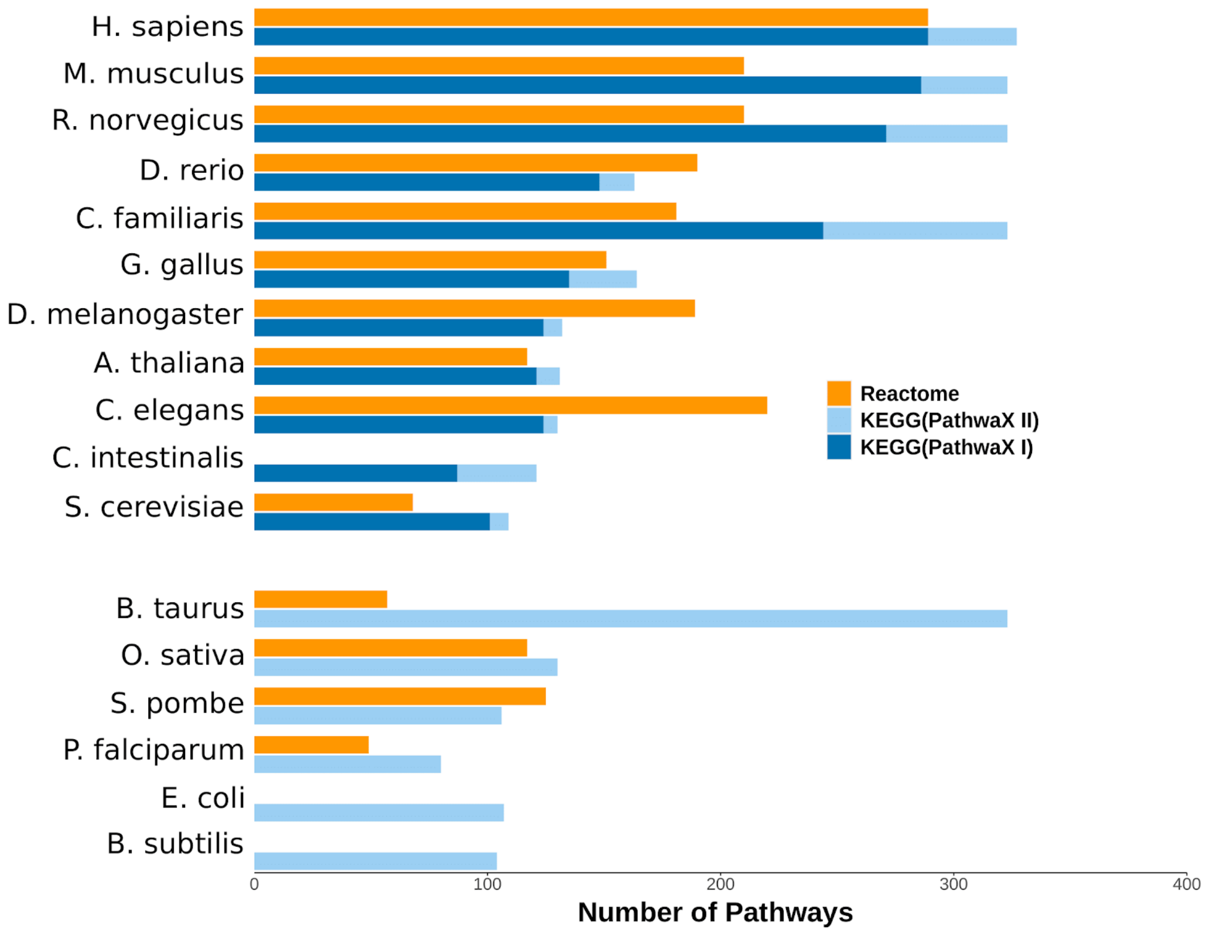


**Figure S2.** Number of pathways per organism in PathwAX II. Pathways were sourced from KEGG v94.1 and Reactome v74, and for comparison the number of KEGG v70.0 pathways used in PathwAX I are shown. Organisms in the top part of the image were already present in PathwAX I, while the other six species in the lower part are new species added to PathwAX II.

**A.**

KEGG v94.1 pathways with crosstalk to the query

| Pathway class | Significant pathways |
|---|---|
| Cellular Processes | 2 |
| Environmental Information Processing | 1 |
| Human Diseases | 15 |
| Organismal Systems | 4 |

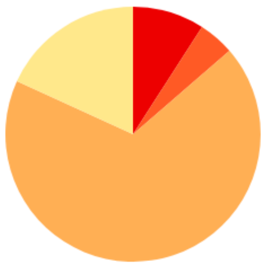

| # | Pathway (enriched / depleted ) | q-value (FWER) | Network connectivity of query genes (pathway gene) |
|---|---|---|---|
| 1 | Systemic lupus erythematosus | 2.6256e-83 | |
| 2 | Staphylococcus aureus infection | 1.1542e-28 | |
| 3 | Alcoholism | 1.9854e-28 | |
| 4 | Viral carcinogenesis | 8.3064e-19 | |
| 5 | Necroptosis | 9.3551e-16 | |
| 6 | NOD-like receptor signaling pathway | 3.2819e-11 | |
| 7 | Phagosome | 7.6980e-9 | |
| 8 | Transcriptional misregulation in cancer | 1.2729e-6 | |
| 9 | Type I diabetes mellitus | 7.2106e-6 | |
| 10 | Asthma | 5.4481e-5 | |
| 11 | Rheumatoid arthritis | 2.8598e-4 | |

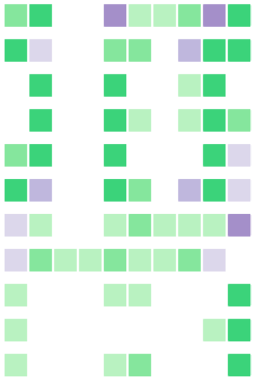

**B.**

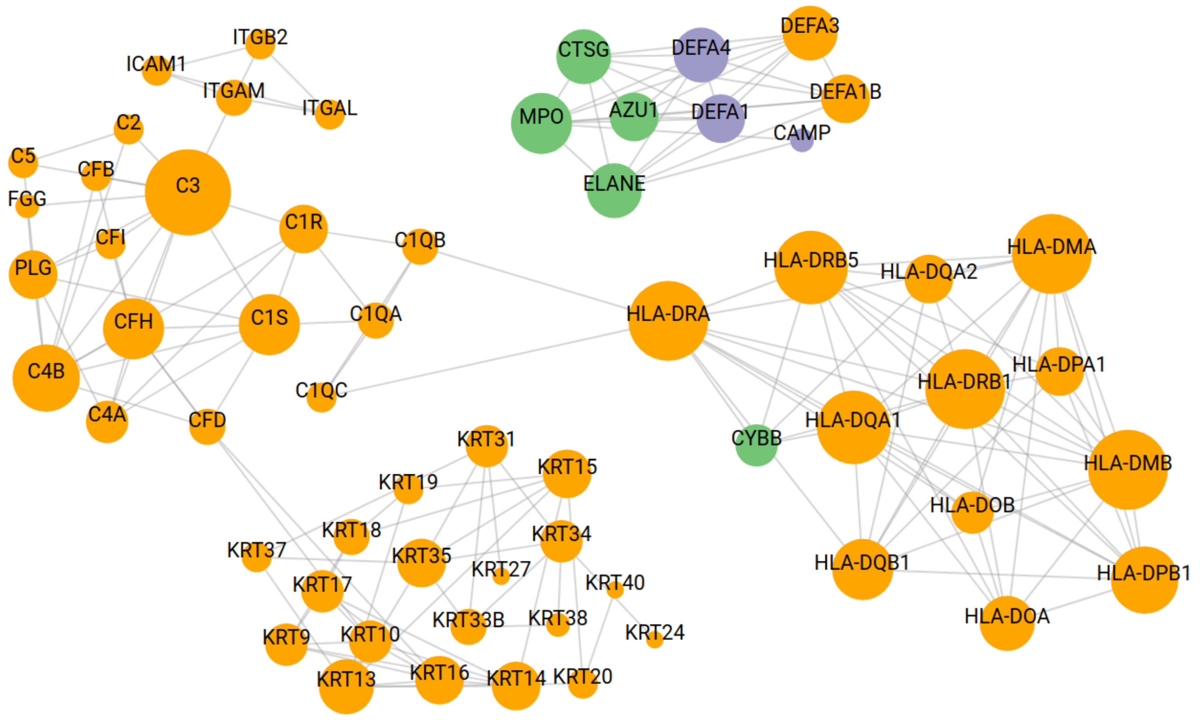


**Figure S3.** PathwAX II results for the 11 genes in the human gene set MARTINELLI_IMMATURE_NEUTROPHIL_UP in MSigDB. **(A)** List of significant pathways; for explanations see Figure S1. **(B)** Network visualization of the crosstalk with the *Staphylococcus aureus infection* pathway. Note that the pathway has a distinct modular structure and that one module contains most of the observed crosstalk.

**Table S1.** Coverage of genes in networks and pathways per organism in PathwAX II. The new six species added in PathwAX II are placed at the bottom of the table. Missing species in Reactome are marked as “–”.

| Species | Genes | Links | Unique KEGG genes | Unique Reactome genes |
|---|---|---|---|---|
| *H. sapiens* | 12 051 | 1 222 749 | 7 423 | 7 896 |
| *M. musculus* | 12 105 | 1 309 394 | 7 836 | 6 485 |
| *R. norvegicus* | 11 979 | 1 534 726 | 7 826 | 6 531 |
| *D. rerio* | 8 723 | 757 523 | 4 985 | 5 017 |
| *C. familiaris* | 10 189 | 665 143 | 6 405 | 5 935 |
| *G. gallus* | 6 327 | 284 502 | 3 301 | 3 099 |
| *D. melanogaster* | 6 420 | 444 885 | 2 679 | 2 862 |
| *A. thaliana* | 12 035 | 1 465 923 | 4 748 | 3 156 |
| *C. elegans* | 6 828 | 674 507 | 2 405 | 3 128 |
| *C. intestinalis* | 3 783 | 314 715 | 1 373 | – |
| *S. cerevisiae* | 4 901 | 495 380 | 1 082 | 1 063 |
| *B. taurus* | 11 988 | 1 167 678 | 6 578 | 2 910 |
| *O. sativa* | 5 620 | 845 755 | 3 220 | 2 166 |
| *S. pombe* | 2 441 | 73 698 | 1 319 | 1 164 |
| *P. falciparum* | 1 191 | 36 806 | 791 | 453 |
| *E. coli* | 2 860 | 49 736 | 1 449 | – |
| *B. subtilis* | 2 680 | 12 573 | 1 165 | – |

## REFERENCES


Ashburner,M. *et al.* (2000) Gene Ontology: tool for the unification of biology. *Nature Genetics*, **25**, 25–29.

Bennett,L. *et al.* (2003) Interferon and granulopoiesis signatures in systemic lupus erythematosus blood. *J. Exp. Med.*, **197**, 711–723.

Bostock,M. *et al.* (2011) $D^3$: Data-Driven Documents. *IEEE Trans. Vis. Comput. Graph.*, **17**, 2301–2309.

Chowdhury,S. and Sarkar,R.R. (2015) Comparison of human cell signaling pathway databases—evolution, drawbacks and challenges. *Database*, **2015**.

Croft,D. *et al.* (2014) The Reactome pathway knowledgebase. *Nucleic Acids Research*, **42**, D472–D477.

Domingo-Fernández,D. *et al.* (2019) ComPath: an ecosystem for exploring, analyzing, and curating mappings across pathway databases. *NPJ Syst Biol Appl*, **5**, 3.

Ekman,A.-K. and Cardell,L.O. (2010) The expression and function of Nod-like receptors in neutrophils. *Immunology*, **130**, 55–63.

Heinzelmann,M. *et al.* (1998) Heparin binding protein (CAP37) is an opsonin for Staphylococcus aureus and increases phagocytosis in monocytes. *Inflammation*, **22**, 493–507.

Huang,D.W. *et al.* (2009) Systematic and integrative analysis of large gene lists using DAVID bioinformatics resources. *Nat. Protoc.*, **4**, 44–57.

Jassal,B. *et al.* (2019) The reactome pathway knowledgebase. *Nucleic Acids Research*.

Kanehisa,M. *et al.* (2016) KEGG as a reference resource for gene and protein annotation. *Nucleic Acids Research*, **44**, D457–D462.

Liberzon,A. *et al.* (2011) Molecular signatures database (MSigDB) 3.0. *Bioinformatics*, **27**, 1739–1740.

Mackey,J.B.G. *et al.* (2019) Neutrophil Maturity in Cancer. *Front. Immunol.*, **10**, 1912.

Martinelli,S. *et al.* (2004) Induction of genes mediating interferon-dependent extracellular trap formation during neutrophil differentiation. *J. Biol. Chem.*, **279**, 44123–44132.

McKay,M.S. *et al.* (1999) Immunomagnetic recovery of human neutrophil defensins from the human gingival crevice. *Oral Microbiol. Immunol.*, **14**, 190–193.

Mi,H. *et al.* (2017) PANTHER version 11: expanded annotation data from Gene Ontology and Reactome pathways, and data analysis tool enhancements. *Nucleic Acids Res.*, **45**, D183–D189.

Mubeen,S. *et al.* (2019) The Impact of Pathway Database Choice on Statistical Enrichment Analysis and Predictive Modeling. *Front. Genet.*, **10**, 1203.

Ogris,C. *et al.* (2017) A novel method for crosstalk analysis of biological networks: improving accuracy of pathway annotation. *Nucleic Acids Research*, **45**, e8–e8.

Ogris,C. *et al.* (2018) FunCoup 4: new species, data, and visualization. *Nucleic Acids Research*, **46**, D601–D607.

Rodchenkov,I. *et al.* (2020) Pathway Commons 2019 Update: integration, analysis and exploration of pathway data. *Nucleic Acids Res.*, **48**, D489–D497.

Salemme,R. *et al.* (2019) The Role of NETosis in Systemic Lupus Erythematosus. *J Cell Immunol*, **1**, 33–42.

Slenter,D.N. *et al.* (2018) WikiPathways: a multifaceted pathway database bridging metabolomics to other omics research. *Nucleic Acids Res.*, **46**, D661–D667.

Türei,D. *et al.* (2016) OmniPath: guidelines and gateway for literature-curated signaling pathway resources. *Nat. Methods*, **13**, 966–967.
